# Delivering High-Quality Family Planning Services in Crisis-Affected Settings I: Program Implementation

**DOI:** 10.9745/GHSP-D-14-00164

**Published:** 2015-03-02

**Authors:** Dora Ward Curry, Jesse Rattan, Jean Jose Nzau, Kamlesh Giri

**Affiliations:** a CARE-USA, Atlanta, GA, USA.

## Abstract

In 2012, about 43 million women of reproductive age experienced the effects of conflict. Provision of basic sexual and reproductive health services, including family planning, is a recognized right and need of refugees and internally displaced people, but funding and services for family planning have been inadequate. This article describes lessons learned during the first 2.5 years of implementing the ongoing Supporting Access to Family Planning and Post-Abortion Care in Emergencies (SAFPAC) initiative, led by CARE, which supports government health systems to deliver family planning services in 5 crisis-affected settings (Chad, Democratic Republic of the Congo, Djibouti, Mali, and Pakistan). SAFPAC's strategy focuses on 4 broad interventions drawn from public health best practices in more stable settings: competency-based training for providers, improved supply chain management, regular supervision, and community mobilization to influence attitudes and norms related to family planning. Between July 2011 and December 2013, the initiative reached 52,616 new users of modern contraceptive methods across the 5 countries (catchment population of 698,053 women of reproductive age), 61% of whom chose long-acting methods of implants or intrauterine devices. Prudent use of data to inform decision making has been an underpinning to the project's approach. A key approach to ensuring sustained ability to train and supervise new providers has been to build capacity in clinical skills training and supervision by establishing in-country training centers. In addition, monthly supervision using simple checklists has improved program and service quality, particularly with infection prevention procedures and stock management. We have generally instituted a “pull” system to manage commodities and other supplies, whereby health facilities place resupply orders as needed based on actual consumption patterns and stock-alert thresholds. Finally, reaching the community with mobilization efforts appropriate to the cultural context has been integral to meeting unmet family planning needs rapidly in these crisis-affected settings. Despite the constraints in crisis-affected countries, such as travel difficulties due to security issues, in our experience, we have been able to extend access to a range of contraceptive methods, including long-acting reversible contraceptives, in such settings using best practice approaches established in more stable environments.

## Abstract (precis)

Extending access to a wide variety of contraceptive methods, including long-acting reversible methods, is feasible in crisis-affected countries by focusing on best practices such as competency-based training, supply chain support, systematic supervision, and community mobilization. Prudent use of data helps drive program improvements.

## INTRODUCTION

In 2012, an estimated 61 million people needed humanitarian assistance in response to conflict and natural disasters, and about 43 million women of reproductive age experienced the effects of conflict.^1^

The Programme of Action of the International Conference on Population and Development recognizes the specific sexual and reproductive health (SRH) rights and needs, including family planning, of refugees and internally displaced persons (IDPs).^2^ The *Humanitarian Charter and Minimum Standards in Humanitarian Response*, established by the Sphere Project (a global network of humanitarian agencies), requires provision of basic SRH services, known collectively as the minimum initial service package (MISP), during the acute phase of an emergency.^3^ The MISP recommends immediate delivery of basic emergency obstetric care, prevention and management of sexual violence, and prevention of transmission of HIV and sexually transmitted infections (STIs), to be followed by comprehensive reproductive health services, such as family planning, as soon as the situation stabilizes.

Nonetheless, the provision of family planning services in conflict-affected populations has been inadequate.^4^ Preliminary results from the second global evaluation of the Inter-Agency Working Group on Reproductive Health in Crises show that official development assistance (ODA) disbursement for SRH to 18 conflict-affected countries increased by 298% between 2002 and 2011.^5^ However, overall ODA funding increased as well during this time period, and 56% of the increase in SRH funding was driven by HIV funding.^5^ When taking these factors into account, it becomes apparent that the percentage of funding for non-HIV SRH did not change substantially between 2002 and 2011.

When family planning services are available in such settings, they generally are limited to oral contraceptives and condoms, even though a broad choice of methods is an essential component of good family planning programming.^6^^,^^7^ Moreover, recent research in the humanitarian context, while not extensive, shows it is feasible to increase access to and use of a wide range of family planning methods including highly effective long-acting reversible contraceptives (LARCs).^8^^,^^9^

Family planning services in conflict settings, when available, are usually limited to provision of oral contraceptives and condoms.

The situation may be changing. The global Family Planning 2020 (FP2020) initiative aims to expand access to family planning information, services, and supplies to an additional 120 million women and girls in the world's 69 poorest countries, many of which are experiencing crises, by the year 2020.^10^ The FP2020 initiative explicitly recognizes that women and girls who live in or must flee from conflict zones, or who are displaced by natural disasters, have especially acute family planning needs. In May 2013, the United Nations Population Fund (UNFPA) established a fund to work with the International Planned Parenthood Federation (IPPF) to help an estimated 22 million women in territories emerging from conflicts and natural disasters gain access to family planning services.^11^

CARE's ongoing Supporting Access to Family Planning and Post-Abortion Care in Emergencies (SAFPAC) initiative attempts to meet that need for family planning in 5 crisis-affected countries—Chad, the Democratic Republic of the Congo (DRC), Djibouti, Mali, and Pakistan—through 4 broad intervention areas: competency-based family planning training, supply chain management, facility and provider supervision, and community mobilization. The countries in which SAFPAC operates face persistent challenges, including recurrent natural disasters, chronic conflict, and significant populations of IDPs or refugees.

The purpose of this article is to provide a detailed description of the program's activities, demonstrating that it is possible to deliver high-quality family planning services, including LARCs, in crisis-affected settings using a set of best practices established in more stable settings. This article also distills lessons learned from implementation during the first 2.5 years of the program (July 2011–December 2013). A companion article, also published in *Global Health: Science and Practice*, presents findings from program monitoring data on the effects of the program on contraceptive use.^12^ See also the Box in this article for key findings on modern method use by country.

**BOX. Modern Method Use Among SAFPAC Facility Clients: Country Snapshots**

Despite the challenging environments in the 5 countries in which SAFPAC operates, in the first 2.5 years of the project (July 2011 through December 2013), SAFPAC and its government partners reached 52,616 new contraceptive users, significantly exceeding the target of 36,002 new users over the corresponding project period (Table). Of those new users, 61% chose and received a long-acting reversible contraceptive (LARC).

Choice of method varied by country. In Chad, the Democratic Republic of the Congo, and Mali, the method mix was heavily weighted toward LARCs, particularly implants. In Pakistan, the method mix was balanced between oral contraceptive pills, injectables, and intrauterine devices (IUDs); during the initial project period, access to implants was limited because only doctors could provide the method. Since then, national policy has been changed to allow Lady Health Visitors to also provide implants. In Djibouti, providers did not receive training in IUD and implant insertion until the end of 2013, so the method mix during the initial project period comprised mainly of oral contraceptives and injectables. In all countries, permanent methods represented a negligible portion of the total method mix.

For a more detailed analysis of the effects of the SAFPAC initiative on contraceptive use, please see the companion article published in *Global Health: Science and Practice*.^12^

**TABLE.** New Modern Method Users Among SAFPAC-Supported Facilities, July 2011–December 2013

**Country**	**Countrywide CPR for Modern Methods^a^**	**No. of SAFPAC Facilities**	**WRA in Catchment Area**	**New Modern Contraceptive Users^b^**	**% of LARCs Among All Modern Methods**
Chad	5%	21	405,984	21,191	72%
DRC	8%	21	123,218	14,869	78%
Djibouti	18%^c^	2	5,040	575	1%
Mali	8%	8	14,210	3,093	51%
Pakistan	22%	27	149,601	12,888	29%
**Total**	**NA**	**79**	**698,053**	**52,616**	**61%**

Abbreviations: CPR, contraceptive prevalence rate; DRC, Democratic Republic of the Congo; LARCs, long-acting reversible contraceptives; NA, not applicable; WRA, women of reproductive age.

a Source of countrywide CPR data: Pakistan 2012–13 Demographic and Health Survey (DHS),^13^ DRC 2013–14 DHS,^14^ Mali 2012–13 DHS,^15^ UNFPA 2011 State of the World's Midwifery (for Djibouti),^16^ and Chad 2010 Multiple Indicator Cluster Survey.^17^

b Modern methods consisted of implants, injectables, IUDs, oral contraceptive pills, tubal ligation, and vasectomy.

c The CPR for modern methods was documented to be much lower in the Djibouti camps where SAFPAC works (5.1%).^18^

## PROGRAM DESCRIPTION

The SAFPAC initiative collaborates closely with provincial, district, and health facility management staff, service delivery staff, and community partners on both overall design and day-to-day implementation of the family planning program. In Djibouti, CARE works with the Office of the United Nations High Commissioner for Refugees (UNHCR) since it is the organization directly responsible for health services for refugees.

In all countries, SAFPAC supports basic first-level health centers, referral health centers providing basic delivery services, and district, regional, and provincial hospitals, which provide services to refugees, host country communities, IDPs, and other crisis-affected residents. We work with mid- and higher-level providers including nurses, medical technicians, Lady Health Visitors (community health workers), and doctors. At the time of publication, the project was providing services at 79 facilities across 8 districts in the 5 countries. The combined catchment population at that time was 2,428,145 people, of whom 698,053 were women of reproductive age.

SAFPAC supports the entire range of contraceptive methods sanctioned by the government in each country, including oral contraceptive pills, injectable contraceptives, implants, intrauterine devices (IUDs), permanent methods (although they are offered only in a few facilities that we support), condoms, and emergency contraception (in some countries).

In our program area in Pakistan, the population is largely stable, with recurring major disruption by flooding and somewhat restricted ability to reach populations with services due to local insecurity. In Djibouti, we serve 2 refugee camp populations, while in Mali we serve communities that are affected by insecurity or that are home to IDPs but have no formal IDP settlements or camps. In both Chad and the DRC, we serve a shifting mixture of host communities, refugee camps, and IDPs simultaneously.

SAFPAC's strategy focuses on 4 broad intervention areas:

1. Providing competency-based training with follow-up clinical assessment and coaching
2. Improving existing supply chain management
3. Conducting facility and provider supervision on a regular basis
4. Mobilizing communities to raise awareness about family planning and to shift norms that block women's access to family planning services

SAFPAC focuses on 4 areas: competency-based training, supply chain management, regular supervision, and community mobilization.

The initiative also conducts activities in the same intervention areas to improve delivery and use of postabortion care (PAC). Provision of family planning is an important part of PAC. However, this article focuses on the initiative's family planning activities only.

### Start-Up Activities: Facility Assessments and Stocking

In each project facility, we first conducted a health facility assessment (HFA) to identify gaps in basic infrastructure. The HFA reviewed the presence and condition of basic elements, such as adequate clean water, electricity, and infection prevention items (autoclaves, hand washing stations) and reviewed the stocks of supplies, medicines, and family planning commodities. In addition, the assessment identified how many health care providers in each cadre served each facility and who was offering family planning services. Program staff conducted the initial HFA in conjunction with staff at Columbia University, our evaluation partner, using a tool they developed (see supplementary materials).

Baseline health facility assessments helped identify gaps in basic infrastructure and in the health information system.

Because the initiative prioritizes using data to drive program improvement, we also assessed the existing health information system and tools in the facilities and supported adaptations where necessary. Consequently, we redesigned family planning registers in Chad and the DRC and implemented a new PAC register in all settings. In addition to creating a monthly form for compiling data and reporting up to district and program levels, we also developed wall charts showing key indicators and oriented facility and district management teams on definitions and ways of using the indicators for decision making.

During the early phases of the project, we also reequipped facilities with supplies and equipment, including an initial supply of family planning commodities. Thereafter, the project has funded and procured only those family planning commodities that are part of the government-approved method mix but that are not provided routinely. For commodities supplied by the government, program staff have worked with facility staff and district-level counterparts to strengthen both accountability mechanisms and supply chain management in order to ensure a steady supply of those items.

### Competency-Based Training: Building Clinical Capacity In-Country

Training of health care providers was competency-based, using training materials developed by Jhpiego. The training included both didactic theory sessions and practicums using anatomical models and actual clients under the supervision of an experienced clinician. In a second cohort of training, providers received in-depth family planning counseling training adapted from the Population Council's “Balanced Counseling Strategy Plus (BCS+)” strategy^19^ and the World Health Organization's (WHO's) “Decision-Making Tool for Family Planning Clients and Providers” (DMT).^20^ In our adapted approach, the provider determines the client's reproductive goals and contraceptive preferences and then presents both advantages and disadvantages of a full range of *relevant* methods, in the context of her expressed preferences, per WHO DMT guidance, and based on local concerns and national reproductive health policies. In line with the BCS+ approach, we also supplied providers with the high-quality, user-friendly BCS+ counseling cards to use with clients. In addition, providers received the “WHO Medical Eligibility Criteria Wheel”—a quick reference tool to rule out medical contraindications for specific methods—as well as copies of *Family Planning: A Global Handbook for Providers*.^21^^,^^22^

In the initial phase, cohorts of 15–20 providers received clinical training for approximately 3 weeks focused on the 3 main clinical skills (PAC and insertion of implants and IUDs). Two weeks were devoted to clinical practicum experiences.

Providers receiving training have had a range of backgrounds, comprising mostly doctors, nurses, and midwives, and in Pakistan, Lady Health Visitors. As of June 2014, 304 providers had received training in at least 1 clinical family planning topic. Currently, 227 providers are serving in SAFPAC-supported posts within the government system. Turnover accounts for some of the discrepancy between the number trained and the number still serving in their post. In addition, some clinicians received training only to ensure buy-in and consistency with and from higher (non-practicing) levels of the government health systems and health care provider partners.

Once trained, providers receive a formal skills assessment at 2 opportunities: within 3 months of initial training and, subsequently, at least once a year. A clinical supervisor observes the provider performing the procedure in question and scores the provider's performance using a standardized checklist for each specific skill the project supports: IUD insertion and removal, implant insertion and removal, manual vacuum aspiration (as the preferred method for PAC), and family planning counseling using our adapted DMT/BCS+ counseling approach. Based on the provider's score, the clinical supervisor determines whether the provider is competent. If a provider receives a failing score, the clinical supervisor recommends an appropriate follow-up action, which can include onsite mentoring from a more experienced provider, visits to a facility both staffed by experienced providers and with a higher client volume for the provider to improve confidence and experience, and retraining if necessary.

In the first training phase, professional trainers from regional training institutes (outside the countries) conducted the training workshops. Subsequently, the project supported the establishment of 1 training center in the DRC and 2 training centers in Chad (1 in the national capital and the other in the regional capital nearest our program sites). In each of these training centers, CARE staff first coordinated with management and clinical staff at a large referral facility (with a high client load) to establish a commitment to the idea of a training center and to maintain staff capacity. This included commitments to supervise trainee clinical experience and ensure adequate supplies and materials to meet minimum quality standards. If the training facility's client volume was below the level needed to provide practicum opportunities to each trainee, program and clinic staff carried out intensive social mobilization to increase volume during the training period. Now, with both national professionals prepared as trainers and training centers established, the project can reliably offer high-quality competency-based training both for new providers and for refresher events in settings closer to trainees' home facilities. This minimizes cost and disruption to service delivery at home facilities and makes it easier to offer ongoing follow-up support to providers.

The project helped establish training centers in the DRC and Chad to make it easier to train new providers and provide ongoing follow-up support.

### Supervision and Quality Improvement: Consistent Support to Facilities and Their Communities

Program staff have continued to engage with both facility teams and community leaders to ensure the facility staff's commitment and capacity to maintain a clean, efficient, and respectful environment. Program staff or, whenever possible, district or zonal staff from the government conduct routine supervisory visits on a monthly basis. The facility supervision team may or may not include the clinician who conducts skills assessments and follow-up support, depending on the country. These facility supervision teams assess general clinic conditions, including infection prevention procedures and supplies, check stock levels, and collect data for program reporting as well as assess data consistency across forms, registers, and reports. For general facility supervision, we use a 2-page checklist co-designed by CARE and Columbia University (see supplementary materials).

The facility supervision teams also review and analyze the data displayed on the wall charts with the facility teams and community partners, including informal and formal leaders. This tripartite group (CARE, facility staff, and community leaders) engages in animated discussion of the underlying causes of upward and downward trends and develop action plans together based on those trends.

### Supply Chain: Guaranteeing Availability of a Full Range of Contraceptive Choice

The SAFPAC initiative provides support for procurement, distribution, and management of family planning commodities and other related supplies and equipment. The initiative supplies both long- and short-acting family planning commodities, antibiotics, and pain medicine, where needed, as well as infection prevention supplies such as high-level disinfectant and sterile gloves, where needed. For any supplies and commodities already included in the government's budget and work plan, the initiative provides training in supply chain management directly to the government supply chain system with follow-up review and mentoring in stock management.

SAFPAC has transferred stock to the government system at the highest level practical, for example, supplying a district-level warehouse and then working within the government distribution system. In all countries, the national-level supply chain has not been reliably connected to facilities, either due to decentralization, as in Pakistan and the DRC, or to lack of resources to ensure timely delivery, as in Chad, Djibouti, and Mali. In addition, in Chad, Mali, and Pakistan, security issues sometimes have prevented transport between the provincial-level warehouses and those at the district level, requiring the project to coordinate directly with the districts.

Acquiring contraceptive methods and ensuring their timely delivery to each facility has occupied a major portion of the team's time. Some global-level issues have contributed to supply chain management challenges. For example, high global demand for Jadelle implants that exceeded supply meant the program experienced significant stock-outs for several months. Equally problematic, however, have been “last mile” issues—problems that have prevented commodities and supplies already procured and stored at the district or regional level from being transported to primary care facilities regularly and from being tracked systematically to avoid gaps in availability of the method or treatment.

The project information system tracks key commodities and supplies for each facility and facilitates stock forecasting and logistics management to avoid stock-outs. In general, the project uses a “pull” system, in which facilities estimate monthly average needs and have stock-alert thresholds based on those averages that dictate reordering. In situations where distance, insecurity, or impassable roads during the rainy season mean a delay of one or more months for resupply, the program activates a short-term “push” system, providing several months of stock for one facility. Stock-outs remained a problem until we began reviewing this information system in detail on a monthly basis. The review and feedback to the country teams have successfully reduced stock-outs to nearly zero, which used to occur especially frequently for items that the government systems have committed to supply.

Using a “pull” supply system, facilities estimate monthly average needs and reorder supplies when they reach stock-alert thresholds based on those averages.

### Community Mobilization: Communities as Partners, Not Passive Recipients

Our interventions at the community level include awareness-raising via radio, participatory theater and songs, and large mixed-group dialogue about barriers to women accessing family planning and actions to reduce those barriers. We also work closely with religious leaders across Christian and Muslim faiths not only to raise awareness about services, clarify myths, and share information about the benefits of family planning for women and their families but also to reinforce a woman's right to access health services.

In Chad, the community committees consist of religious and other formal leaders, such as the police, to reinforce a woman's right to time, space, and limit births, using the little known Chadian reproductive health law that ensures this right. As a result, in Chad (as well as the DRC), religious leaders across faiths are dynamic champions of healthy birth spacing and limiting, and in Chad, they even go house-to-house to counsel couples directly. As discussed earlier, religious leaders also serve on our health committees together with the facility staff, representing the community in reviewing the data and in activity planning.

Religious leaders in the refugee camps in Chad and Djibouti have proved to be among the health facility teams' strongest allies, organizing group meetings and conducting one-on-one household visits with couples to promote birth spacing through modern contraception. In the 2 Djiboutian refugee camps where we work, new family planning users have increased markedly after our community leader training, despite a very conservative religious context and fertility norms that support very large families.

Religious leaders have been among the project's strongest community allies in some countries.

## LESSONS LEARNED

Many women in crisis-affected settings have an acute, unmet need for family planning and very little access to services. Despite the many constraints in these settings, the approach used by the SAFPAC initiative has succeeded in making services available to severely underserved communities in challenging settings using relatively straightforward information and service strengthening interventions. In particular, routinely collecting reliable and relevant data and using that data to improve quality at the facility and program levels was an underpinning to the “4 pillars” of the project's approach.

Prudent use of data to inform decision making has laid a strong foundation for the project.

The initiative has faced some challenges that are unique to fragile, crisis-affected countries, such as travel constraints due to security issues making supervisory visits difficult. However, overall the challenges have been similar to those faced by any health or development program, such as ensuring systematic supervision, reaching the community with mobilization efforts appropriate to the cultural context, managing commodities and other supplies to avoid stock-outs, and ensuring accountability. Several lessons learned have emerged in these areas.

### Competency-Based Training

The dearth of in-country clinical experts for training, ongoing coaching, and follow-up supervision has made continuing to support trainees difficult. Few, if any, providers have possessed adequate expertise with both training and implant and IUD insertion and removal to train new providers in the 5 countries. Initially, trainers were brought in from foreign countries or providers were sent out to foreign countries. Realizing that the more lasting solution to this problem is to develop in-country capacity to train clinicians, SAFPAC has started developing a cadre of trainers in Chad, the DRC, and Pakistan that is equipped to train new health workers to provide a full range of contraceptive services.

Low patient volumes in some of the smaller facilities have made it challenging for clinical mentors to provide ongoing coaching and skills assessment. Providers remain reluctant to perform procedures with which they are uncomfortable, especially those requiring pelvic examinations, such as IUD insertion. The program has addressed this barrier by using anatomical models for skills demonstration during routine supervisory visits and in skills refresher sessions.

Tracking the skills assessments performed has been one of the most significant challenges with project monitoring, largely due to the difficulty of coordinating availability of clinical supervisors, trainees, and clients for each service. The lack of centralized personnel records has made maintaining records of each provider's training, assessment, follow-up history, and current status extremely difficult. We have met this challenge by developing a training and performance tracker that uses a Microsoft Excel datasheet to record the date of last assessment, its outcome, and follow-up steps planned for each provider and for each skill.

### Supervision and Quality Improvement

We believe strengthening and fine-tuning supervision has been a key strategy of the program. The supervision staff fills 2 major functions: recurring, systematic feedback and problem solving. Key elements to our supervision approach include monthly checklists, regular site visits, and meetings with community leaders, clinic staff, and CARE facilitators to review progress.

Conducting monthly supervisory visits to every facility has been challenging. In the early stages of the program, road and security conditions and inadequate logistical arrangements sometimes contributed to missed supervision visits. In addition, guidance from program leadership had not adequately emphasized that a supervisory visit to every facility every month was a critical input, nor had it required data from supervisory checklists to be reported at the country or global level. Hiring additional supervisory staff and revising the supervisory protocol have been key approaches to achieving that standard.

Each SAFPAC supervisor now covers 5–9 facilities in the African countries (with the exception of Djibouti, where we cover only 2 facilities). In Pakistan, because of the much shorter distances and better infrastructure, 1 supervisor is responsible for general facility supervision for approximately 10–12 facilities, with 2 clinical supervisors covering assessments and coaching for approximately 13 facilities. Manageable workloads have made it feasible for supervisors to conduct thorough site visits on a regular basis.

Hiring additional staff to make supervision workloads manageable has helped ensure regular site visits.

The improvement in infection prevention procedures after the introduction of a simplified monthly supervisory checklist serves as an excellent example of the power of ongoing systematic feedback. Infection prevention had been a persistent problem. Initially, supervisors were supposed to use a lengthy checklist to assess adherence to infection prevention procedures and other issues, but they were not required to report the data to a higher level. The new 2-page checklist has replaced the lengthier one, and supervisors use the new one consistently. In addition, the new checklist enables supervisors to calculate a simple score that focuses their attention on infection prevention. Within 3 months after introduction of this new checklist, average scores increased significantly and infection prevention practices improved markedly.

In addition to consistent feedback from supervisors, flexibility in the supervisory approach also has been a key element contributing to our success. For example, in the DRC, program staff and zonal teams have resolved persistent stock management issues using a “pull system” that uses average stock levels and alert thresholds to stabilize stock levels, while in Chad, teams have chosen to push larger amounts of stock to the facilities and purchase some consumables locally to minimize stock-outs due to impassable roads, insecurity, or sudden increases in clients from escalating refugee populations. Stock-outs are now a rare occurrence in both countries.

### Supply Chain

In terms of procurement, one of the biggest challenges for the program as a whole has been the difficulty of obtaining Jadelle implants, which have become so popular with family planning programs that demand often outstrips supply. (See companion article in *Global Health: Science and Practice* for more information about the popularity of implants in these settings.^12^) This has resulted in some pipeline breaks in Chad and the DRC. We have been responding to this is by creating buffer stocks in each country program, loaning stocks between countries, ensuring all providers are trained in inserting and removing both Jadelle and Implanon implants so that they can substitute for each other, and building relationships with UNFPA and other large stock holders.

At the other end of the spectrum are the “last mile” issues—getting the contraceptives and related medical supplies to health facilities where they are needed and when they are needed. Health facilities in some countries continue to experience periodic stock-outs of contraceptives and medical supplies (e.g., pain medicine, gloves, high-level disinfectant) even when these items are available either in the warehouse or pharmacy that resupplies them or in the market place. These stock-outs are typically site-specific and of relatively short duration; however, our aim is to prevent all stock-outs in order to ensure high-quality service. In order to address this problem, the initiative has instituted a “pull” system as the general norm, as mentioned previously, whereby heath facilities place resupply orders as needed based on actual consumption patterns.

The monthly reports received by district health officials and CARE staff include some stock-related data for key commodities and related medical supplies. Having access to these data and providing feedback consistently have also been key approaches to resolving persistent problems with stock management. Regular reviews of such data every 6 weeks supported the country teams in: (1) solving problems with repeated stock-outs, (2) forecasting need to avoid stock-outs, and (3) determining how to advocate with local systems to ensure they provide basic supplies and medications (per agreements established at the beginning of the project) and also to ensure clinic conditions meet a minimum standard of quality at all times.

### Community Mobilization

A deliberate evidence-based community mobilization strategy is integral to meeting unmet family planning needs rapidly in crisis-affected settings. Simply raising basic awareness of new and underused methods among community members was a necessary first step in the settings where we work. In addition, in most settings, the key to successful community mobilization has been engaging and inspiring the whole community to consider the benefits and opportunities inherent not only in planning and spacing births but also in clearing the path for women to use services.

Ensuring women's right to access health services has been a key component of community mobilization efforts.

Specifically, each country program has incorporated a context-specific strategy to develop champions among influential men in the community, most often religious leaders and municipal authorities. In Pakistan, we have focused on the message that well-spaced pregnancies lead to healthy children, and we have mobilized municipal and health system leadership to agree that ensuring women's access to services was the expression of this theme. In Chad and the DRC, religious leaders across faiths have been dynamic champions of healthy birth spacing and limiting, going house-to-house to counsel couples directly. In Chad, there was initial resistance among men when they realized women were increasingly using family planning, resulting in several husbands filing complaints at the local police station. CARE has supported religious leaders and police in learning about and ensuring the application of a little-known national reproductive health law affirming women's right to use family planning, with or without their partner's permission. In the 2 Djiboutian refugee camps where we work, new family planning users have increased markedly after our community leader training, despite a very conservative religious context and fertility norms that support very large families.

### Country-to-Country Variations

Some clear differences between countries have emerged, the most variable of which have been: (1) context set by the national government health system, (2) community norms related to gender, fertility, contraception, and community members' comfort level in talking publicly about these issues, and (3) the regularity of payment to government health workers.

#### National Government Health Systems

The strength of the national government health system has had an unexpected impact on our programming. The government health system in Chad was possibly the most over-taxed among SAFPAC countries before our program began. There, our efforts have been met with interest and enthusiasm. The program staff has collaborated directly with the ministry of health at district and national levels to support enhanced guidelines and has even reformulated policy. For example, the Chadian government has sanctioned the new family planning registers we designed at the national level. The government has also approved lower-level cadres of health workers (nurses and midwives) to provide implants and IUDs as well as guidance that allowed this service to be provided at the health center level. This guidance was eventually adopted as national family planning policy. In a very different context, in Pakistan's large, decentralized health system, obtaining approval for piloting new registers and authorization for Lady Health Visitors to insert implants proved to be a lengthy process, which required involvement from multiple levels of the health system.

#### Community Norms

In our experience, successful approaches to community mobilization require more adaptation and flexibility than do those aimed at improving clinical quality. Our mobilization has relied most heavily on committees of interdenominational religious leaders in Chad, women's associations and health committees focused on social accountability in the DRC, and formal leaders, religious leaders, and various camp committees (such as youth and sport) in refugee-only programming in Djibouti. In Pakistan, we have worked with village health committees and Lady Health Visitors, who are more formally integrated into the health system than in other countries.

#### Compensation of Health Workers

The DRC provides a dramatic illustration of the significant and context-specific obstacle posed by inadequate, irregular, and missing compensation of health workers. In the other countries, frontline health workers are compensated more reliably. In the DRC, some health worker cadres receive only modest wages drawn from the nationally sanctioned system of cost-recovery, in which most services including family planning are at a cost to clients. However, the SAFPAC approach requires that family planning services be provided free of charge (in all countries). As a result, providers forego income by providing family planning services when they could be charging for curative care. The fact that we have still seen sustained, high levels of new users each month for more than 2 years demonstrates the motivational power of regular supervision and training opportunities.

## RECOMMENDATIONS AND NEXT STEPS

SAFPAC has rapidly reached more than 52,000 new family planning users with a wide range of modern methods in diverse crisis-affected settings (see companion article in *Global Health: Science and Practice*^12^). Based on our experience, we recommend 3 strategies for establishing more comprehensive family planning services in such settings:

1. Prioritize building clinical skills training and supervision capacity to ensure continued ability to train and supervise new providers after the end of a donor-funded project
2. Incorporate selected quality improvement strategies, as feasible, adding additional elements with time
3. Conduct a local participatory situation analysis as early as possible to further refine global best practices in community engagement and social accountability strategies

3 key strategies for delivering family planning services in fragile settings: build capacity in clinical skills training and supervision; incorporate quality improvement approaches gradually; and tailor community engagement efforts to the local context.

While our first recommendation may seem simplistic, it is both more complex and has broader importance than it may seem. Skilled and confident frontline health workers and a minimum level of cleanliness and number of providers are prerequisites to reaching the population with health services and to improving the community's perception of and trust in the health facility. This is also important for the morale of providers. Not only does commitment to building national capacity for clinical supervision and program quality assurance improve results in the near term but it also makes a meaningful contribution to the country's long-term ability to reach refugees, IDPs, and other crisis-affected communities with high-quality family planning services.

The dramatic and rapid improvement of both our infection prevention procedures and stock management after the implementation of supervision checklists and monthly feedback provide clear evidence that many of the quality improvement techniques used in stable and even more developed settings can be effective in these challenging contexts. We are currently piloting client exit interviews as an additional quality assurance strategy and a mechanism to assess client satisfaction with services. In addition, the project plans on strengthening the current tracking system for removals of contraceptive devices and reasons for removal to assess women's satisfaction with their method choice, frequency of side effects, and perceptions of provider competency. This is especially important as implants provided early in the program will eventually lose effectiveness and will need to be removed.

Finally, the importance of community engagement cannot be underestimated and must be carefully tailored to the context. This recommendation does apply especially to health programming related to sex, sexuality, and fertility, such as family planning, as well as to places where contraception is either unknown or feared and where people learn and share primarily through their local social networks. While all CARE's community engagement (and health systems strengthening) interventions take a rights-based approach as the starting point, each community has a preexisting context of norms, beliefs, and community institutions. Real community engagement at the most local level on issues of rights, fertility, and contraception must occur among men and women, across religious and ethnic lines, and between facility staff and community members for family planning to reach those who want it.

## CONCLUSION

The SAFPAC experience so far has been one of iterative learning. Fragile states will not change overnight, but there is space to influence and make change, albeit slowly and incrementally, at the local and even national level. The unfortunate reality is that most crisis situations will not resolve in a matter of a few months but will last many months and often years. Considering these settings and time frames, programs should ensure that minimum services are available (including long-acting family planning methods) and, at the same time, incrementally tackle underlying causes of poor health services. Further, we show that strategies drawn from more stable development settings—competency-based training, supportive supervision, simple quality improvement tools, and community mobilization that works with local power structures while simultaneously exploring restrictive norms—can be modified to work in even these unstable settings. Implementation of quality improvement and clinical supervision through the government health system builds national capacity and can provide data and mobilize support for creating a more supportive policy environment. These approaches can not only lead to significantly expanded access to and use of family planning in a short timeframe but also support longer-term improvements in the fragile health systems themselves.

## Notes

**Peer Reviewed**

**First published online:** 2015 Feb 4
